# Rapid and Objective Assessment of Neural Function in Autism Spectrum Disorder Using Transient Visual Evoked Potentials

**DOI:** 10.1371/journal.pone.0164422

**Published:** 2016-10-07

**Authors:** Paige M. Siper, Vance Zemon, James Gordon, Julia George-Jones, Stacey Lurie, Jessica Zweifach, Teresa Tavassoli, A. Ting Wang, Jesslyn Jamison, Joseph D. Buxbaum, Alexander Kolevzon

**Affiliations:** 1 Seaver Autism Center for Research and Treatment, Icahn School of Medicine at Mount Sinai, New York, New York, United States of America; 2 Department of Psychiatry, Icahn School of Medicine at Mount Sinai, New York, New York, United States of America; 3 Ferkauf Graduate School of Psychology, Yeshiva University, Bronx, New York, United States of America; 4 Hunter College, New York, New York, United States of America; 5 Department of Neuroscience, Icahn School of Medicine at Mount Sinai, New York, New York, United States of America; 6 Friedman Brain Institute, Icahn School of Medicine at Mount Sinai, New York, New York, United States of America; 7 Department of Genetics and Genomic Sciences, Icahn School of Medicine at Mount Sinai, New York, New York, United States of America; 8 Mindich Child Health and Development Institute, Icahn School of Medicine at Mount Sinai, New York, New York, United States of America,9 Department of Pediatrics, Icahn School of Medicine at Mount Sinai, New York, New York, United States of America; 10 Centre for Research in Autism and Education (CRAE), UCL Institute of Education, University College London, London, United Kingdom; Tokai University, JAPAN

## Abstract

**Objective:**

There is a critical need to identify biomarkers and objective outcome measures that can be used to understand underlying neural mechanisms in autism spectrum disorder (ASD). Visual evoked potentials (VEPs) offer a noninvasive technique to evaluate the functional integrity of neural mechanisms, specifically visual pathways, while probing for disease pathophysiology.

**Methods:**

Transient VEPs (tVEPs) were obtained from 96 unmedicated children, including 37 children with ASD, 36 typically developing (TD) children, and 23 unaffected siblings (SIBS). A conventional contrast-reversing checkerboard condition was compared to a novel short-duration condition, which was developed to enable objective data collection from severely affected populations who are often excluded from electroencephalographic (EEG) studies.

**Results:**

Children with ASD showed significantly smaller amplitudes compared to TD children at two of the earliest critical VEP components, P_60_-N_75_ and N_75_-P_100_. SIBS showed intermediate responses relative to ASD and TD groups. There were no group differences in response latency. Frequency band analyses indicated significantly weaker responses for the ASD group in bands encompassing gamma-wave activity. Ninety-two percent of children with ASD were able to complete the short-duration condition compared to 68% for the standard condition.

**Conclusions:**

The current study establishes the utility of a short-duration tVEP test for use in children at varying levels of functioning and describes neural abnormalities in children with idiopathic ASD. Implications for excitatory/inhibitory balance as well as the potential application of VEP for use in clinical trials are discussed.

## Introduction

Significant advances in the field of autism spectrum disorder (ASD) have been made over the past decade, including the identification of approximately 100 causal genes [1, 2], successful preclinical studies demonstrating treatment efficacy in model systems [3, 4], and the first large-scale clinical trials currently underway in patients with single-gene forms of ASD. However, despite these advances, the field continues to rely on behavioral measures for both diagnosis and treatment monitoring. Validated biomarkers of ASD remain limited and there are no accepted markers for widespread use [5]. While electrophysiological methods have improved our understanding of the disorder, these methods are only recently being tested as outcome measures [6, 7] and studies are often biased towards higher functioning individuals.

This study seeks to examine disease pathophysiology by examining putative *γ*-aminobutyric acid (GABA/inhibitory) and glutamatergic (excitatory) activity in children with idiopathic ASD and their unaffected siblings (SIBS) using visual evoked potentials (VEPs). VEPs offer a noninvasive, objective, and reliable technique used to assess the functional integrity of visual pathways and reflect the sum of excitatory and inhibitory postsynaptic potentials occurring on apical dendrites [8]. The major positive and negative peaks and troughs in VEP waveforms are considered a reflection of cortical activity and different cellular events [8, 9].

Pharmacological studies provide evidence for the electrogenesis of these electrically evoked potentials. When GABA-blocking agents are topically applied to the cortex to selectively block GABA_A_-mediated inhibition, there is an enhancement of the negative wave and an attenuation or elimination of the subsequent positive wave. In contrast, when GABA is applied to the cortex, the negative wave is attenuated or eliminated and the subsequent positive wave is enhanced [10, 11].

While tVEPs have been recorded from individuals with single-gene forms of ASD (e.g., fragile X syndrome (FXS) [12] tuberous sclerosis complex [13] Phelan-McDermid syndrome (PMS) [14] Rett syndrome [15, 16], no published studies have examined tVEP responses in children with idiopathic ASD or SIBS. Given the important role balanced excitatory and inhibitory synapses play in healthy brain function [17–20] VEPs provide a method to examine theories of excitatory/inhibitory (E/I) imbalance in ASD [21]. There is considerable evidence, ranging from cellular studies to human studies, supporting theories of enhanced excitation and suppressed inhibition as markers of ASD [21–26] However, our current understanding of single-gene causes of ASD, based in large part on findings from animal models, suggests that glutamatergic activity is heightened in certain syndromes (e.g., FXS) [[27] and decreased in others (e.g., PMS) [28]; these findings highlight the heterogeneity of ASD in which the excitatory and inhibitory balance is variable. Ultimately, subtyping patients based on their E/I profile may inform personalized medical approaches, aid in determining optimal treatment targets and, in turn, predict treatment responders based on an individual’s baseline profile.

The current study applied a standard contrast-reversing checkerboard VEP test [29] and aimed to validate a novel short-duration VEP test, which was developed to optimize objective data collection from difficult to test populations[30]. For both the standard and short-duration tests, we hypothesized that children with ASD would display weaker responses reflecting an altered E/I balance as compared to typically developing (TD) controls and expected SIBS to display an intermediary response. These aims were achieved as described below.

## Materials and Methods

### Participants

One hundred and ten children between the ages of two and 12 participated in this study. Data from 14 participants were removed due to: excessive movement or noncompliance (*n* = 6), failure to meet diagnostic inclusion/exclusion criteria (*n* = 5), or presence of a genetic finding (*n* = 3). The final sample included usable data from 96 unmedicated children: 37 children with ASD (5 females, *M*_*age*_ = 6.46 years, *SD* = 3.19), 36 TD children (16 females, *M*_*age*_ = 5.89 years, *SD* = 2.45), and 23 SIBS (10 females, *M*_*age*_ = 8.00 years, *SD* = 2.34). There was no significant difference in age between the ASD and TD group, *t(71*) = -.855, *p* = .395, however, there was a significant difference in age between the SIBS group relative to the ASD, *t(58*) = -2.00, *p* = .050, and TD groups *t(57*) = -3.289, *p* = .002. Age was therefore taken into account as a covariate.

ASD participants were diagnosed according to a consensus diagnosis determined by Diagnostic and Statistical Manual of Mental Disorders, Fifth Edition (DSM-5) criteria (APA, 2013), a clinical intake with a child and adolescent psychiatrist or licensed psychologist, and standardized assessments including the Autism Diagnostic Observation Schedule, Second Edition (ADOS-2)[31] and the Autism Diagnostic Interview-Revised (ADI-R)[32]. Participants in the ASD group received genetic testing (chromosomal microarray analysis) and were only included if there was no genetic finding. All SIBS included in this study had an affected sibling with idiopathic ASD.

Cognitive functioning was measured in the ASD group using the Stanford-Binet Intelligence Scales, Fifth Edition (SB-5) [33], the Differential Ability Scales, Second Edition (DAS-II) [34], or for minimally verbal children, the Mullen Scales of Early Learning [35]. In order to examine the relationship between IQ and neural responses, this study included children with ASD of varying levels of cognitive functioning. Adaptive functioning was measured using the Vineland Adaptive Behavior Scales, Second Edition (VABS-II), Survey Interview Form [36] (Table 1).

**Table 1:** Characteristics of ASD Participants.

Characteristic	*M (SD)*
Nonverbal IQ	*88*.*92 (25*.*23)*
	Range: 42–140
Verbal IQ	*80*.*22 (23*.*51)*
	Range: 40–122
ADOS-2 Social Affect Domain	*11*.*12 (4*.*17)*
ADOS-2 Repetitive, Restricted Behavior Domain	*4*.*44 (2*.*11)*
ADOS-2 Total Score	*15*.*56 (5*.*70)*
ADOS-2 Severity Score	*7*.*41 (2*.*05)*
ADI-R Social Domain	*18*.*48 (5*.*79)*
ADI-R Communication Domain	*15*.*93 (4*.*92)*
ADI-R RRB Domain	*7*.*04 (2*.*37)*
Vineland-II Adaptive Behavior Composite	*77*.*39 (9*.*26)*

IQ and Vineland-II scores are listed as standard scores. ADOS-2 and ADI-R scores are listed as raw scores.

Participants in the TD and SIBS groups were screened with the Social Responsiveness Scale, Second Edition (SRS-2) [37]. Total scores on the SRS-2 were significantly higher in the ASD group compared to both TD, *p* < .001, and SIBS groups, *p* < .001. There was no significant difference in SRS-2 total scores between the TD and SIBS groups, *p* = .528. Informed written consent was obtained from all caregivers and assent was obtained from children seven years or older when appropriate. The Mount Sinai Program for the Protection of Human Subjects approved the experiments.

### VEP Recording

A Neucodia system (VeriSci. Corp., USA) was used for stimulus presentation and data collection. Gold-cup electrodes were placed on the midline of the scalp based on the International 10–20 system, which includes an active electrode at *Oz* (occipital), a reference electrode at *Cz* (vertex), and a ground electrode at *Pz* (in between *Oz* and *Cz*) [38]. These three electrodes comprised a single electrophysiological channel. All EEG’s were recorded synchronized to the display’s frame rate. The Neucodia system provided automated artifact detection, which determined whether the EEG recording was affected by excessive 60-Hz noise or drift/saturation. For short-duration tests, if an artifact was detected, the EEG epoch was deleted automatically and the examiner was prompted to repeat the run. For the standard single-trial run, the complete 60-second stimulus was repeated if an artifact was present. An operator verification function and an infrared camera with a separate monitor enabled the examiner to monitor gaze fixation and determine whether participants were attending to the screen. The EEG was amplified (gain = 20,000, bandpass filter: .5–100 Hz) and digitized.

### Stimuli

Stimulus field size subtended 10° x 10° of visual angle (viewing distance = 114 cm). Space-average luminance was ~50 cd/m^2^ and the frame rate was 150 Hz. A checkerboard pattern consisting of 32 x 32 checks (check size = 18.75 minarc) was contrast reversed with a 1-Hz square-wave signal (100% contrast). Two versions of the contrast-reversing checkerboard were administered, including a standard stimulus displayed for 60 seconds [29] and a new short-duration condition in which ten three-second (~1 s adaptation and 2-s EEG epoch) runs were obtained [30]. The order of stimulus presentation alternated between subjects.

### VEP Procedures

A visual schedule was used to explain the VEP procedure to all participants. Three surface electrodes were applied to the scalp using water-soluble electrode paste. Participants sat at a viewing distance of 114 cm and were prompted to fixate on a crosshair in the center of the display screen. An auditory signal cued participants prior to each stimulus presentation. An infrared camera was used to ensure that participants were fixating on the screen and a research assistant was present at all times to aid in behavior management. All participants had normal (20/20) or corrected to normal visual acuity at the viewing distance of 114 cm.

### Analysis

#### Time-domain analyses

A discrete Fourier transform was applied to the EEG data to extract harmonic frequency components of the response, and waveforms were reconstructed using even harmonics 2–84 Hz, minus the 60 Hz component. The contrast-reversing checkerboard stimulus used in this study produces a positive peak at approximately 60 ms (P_0_ or P_60_), which reflects activation of the primary visual cortex from the lateral geniculate nucleus. A negative trough at approximately 75 ms (N_0_ or N_75_) reflects depolarization and putative glutamatergic postsynaptic activity spreading to the superficial layers of primary visual cortex, and a positive peak at approximately 100 ms (P_1_ or P_100_) reflects superficial hyperpolarization and putative GABAergic activity [10]. Multivariate analyses of variance (MANOVAs) were used to assess differences among groups for amplitude (peak-to-trough) and latency (peak time).

#### Frequency-domain analyses

A magnitude-squared coherence (MSC) statistic was used to quantitatively assess the integrity of overall responses in different frequency bands. Mean MSC values for each band were calculated based on previous work using principal component analysis, which identified the relevant frequency bands [39]. The six distinct frequency mechanisms include: Band 1, 6–10 Hz; Band 2, 12–28 Hz; Band 3, 30–36 Hz; Band 4, 38–48 Hz; Band 5, 50–64 Hz, minus 60 Hz; Band 6, 66–84 Hz. Band 1 reflects alpha-wave activity, Band 2 reflects beta-wave activity, and Bands 3–6 reflect gamma-wave activity. MSC refers to the reliability of the response and estimates signal power/signal+noise power. MSC was calculated to determine consistency from one trial to the next in both size and at a given frequency. A pure signal would yield a value of 1 and no signal would produce a value about 0.1 (bias level for pure noise given ten EEG epochs). MANOVAS were run to examine Group x Frequency Band interactions. Intraclass correlation coefficients (ICCs) were obtained to assess the reliability of responses between the standard- (60-s) and short-duration conditions.

## Results

All 96 participants in the final sample completed at least one of the two stimulus conditions. The standard condition was completed by 25 participants with ASD, 28 TD participants, and 23 SIBS. The short-duration condition was completed by 34 children with ASD, 30 TD children, and 22 SIBS.

### Time-domain analyses

There were no group differences in the latency of responses at P_60_, N_75,_ or P_100_. Amplitudes were measured from peak to trough, P_60_-N_75_ and N_75_-P_100_ (Table 2). Results indicated a significant main effect by group for amplitude in response to both the standard 60-s contrast-reversing checkerboard condition [F(4,146) = 3.755, *p* = .006] and the short-duration condition [F(4,166) = 5.260, *p* = .001].

**Table 2:** Amplitude and Latency.

	Standard Condition					Short-Duration Condition				
	*Amplitude (μV)*		*Latency (ms)*			*Amplitude (μV)*		*Latency (ms)*		
Group	P60-N75*	N75-P100*	*P60*	*N75*	*P100*	P60-N75*	N75-P100*	*P60*	*N75*	*P100*
ASD	7.75	17.91	53.00	71.40	102.40	9.80	20.01	52.06	71.12	99.94
	(3.66)	(6.79)	(4.87)	(3.30)	(7.70)	(5.59)	(9.33)	(4.85)	(4.30)	(7.57)
TD	15.00	29.62	51.32	69.68	100.46	18.99	33.27	51.40	70.33	100.20
	(8.29)	(15.64)	(5.84)	(3.39)	(7.47)	(11.05)	(15.44)	(6.03)	(3.74)	(8.09)
SIBS	13.15	25.43	51.39	71.13	103.74	16.53	29.95	51.14	69.27	101.68
	(7.91)	(12.56)	(3.46)	(4.03)	(8.18)	(8.55)	(11.66)	(3.20)	(3.88)	(9.13)

Values are presented as mean (standard deviation) for the standard 60-s contrast-reversing checkerboard condition and the short-duration condition consisting of ten trials (3-s each). Asteriks indicate p-values < .05.

For the standard condition, significant univariate main effects were obtained for both P_60_-N_75_ [F(2,74) = 7.586, *p* = .001] and N_75_-P_100_ [F(2,74) = 6.040, *p* = .004] amplitudes. Pairwise comparisons showed that the ASD group had significantly smaller amplitudes than the TD group for P_60_-N_75_ (*p* < .001) and N_75_-P_100_ (*p* = .001). Amplitudes in the ASD group were also significantly smaller compared to SIBS for both P_60_-N_75_ (*p* = .009) and N_75_-P_100_ (*p* = .040).

**Fig 1:**
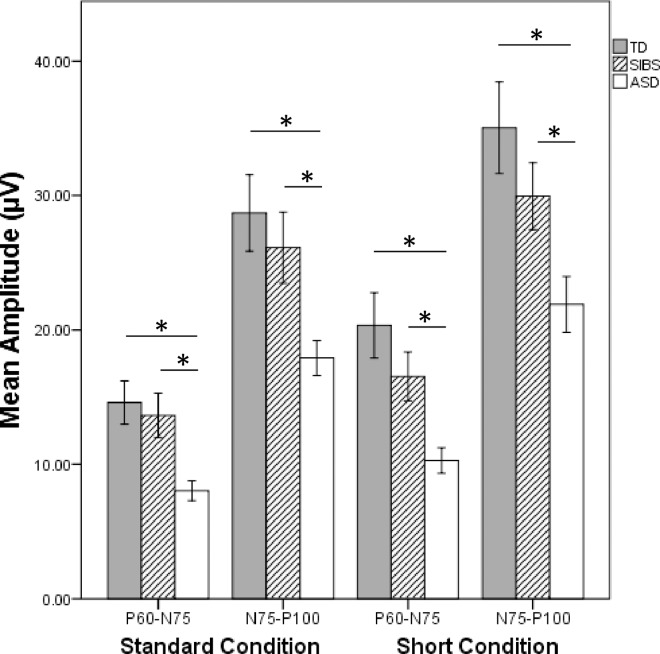
Mean Amplitude by Group. Children in the ASD group showed significantly smaller P_60_-N_75_ and N_75_-P_100_ amplitudes on both the standard condition and the short-duration condtition compared to the TD and SIBS groups. Significance bars indicate p-values < .05. Error bars: +/- 1 *SE*.

**Fig 2:**
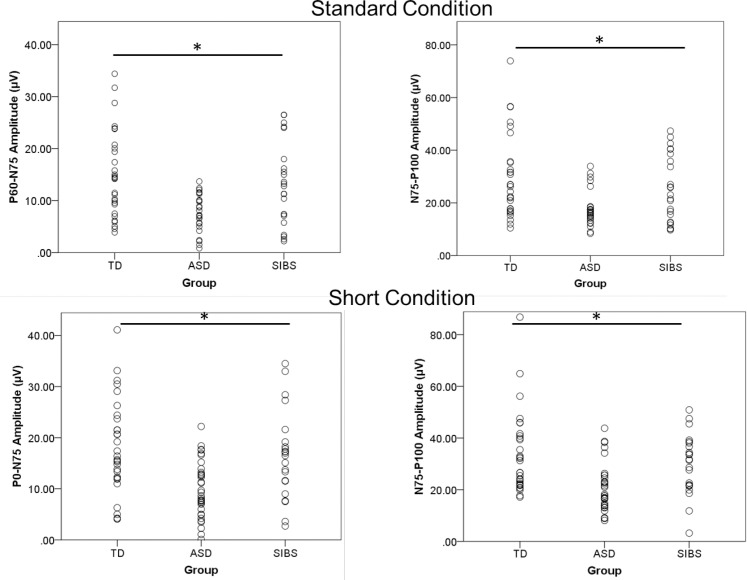
Amplitude by Individual. Scatterplots depict individual amplitude values by group for the standard and short-duration condition. Significance bars indicate p-values < .05.

The short-duration condition produced the same result. Significant univariate main effects for amplitude were obtained for P_60_-N_75_ [F(2,84) = 10.369, *p* < .001] and N_75_-P_100_ [F(2,84) = 9.839, *p* < .001]. Pairwise comparisons indicated that the difference was driven by significantly smaller amplitudes in the ASD group compared to the TD group for P_60_-N_75_ (*p* < .001) and N_75_-P_100_ (*p* < .001). The ASD group also showed significantly smaller amplitudes than the SIBS group for P_60_-N_75_ (*p* = .004) and N_75_-P_100_ (*p* = .005). There were no significant differences in amplitude between TD and SIBS groups, although SIBS did show responses between that of the ASD and TD groups (Figs 1 & 2).

There were no significant Pearson correlation coefficients between amplitude measures and IQ or age in the ASD group (all *p*-values > .05). Furthermore, results persisted when age and sex were taken into account as covariates.

### Frequency-domain analyses

**Fig 3:**
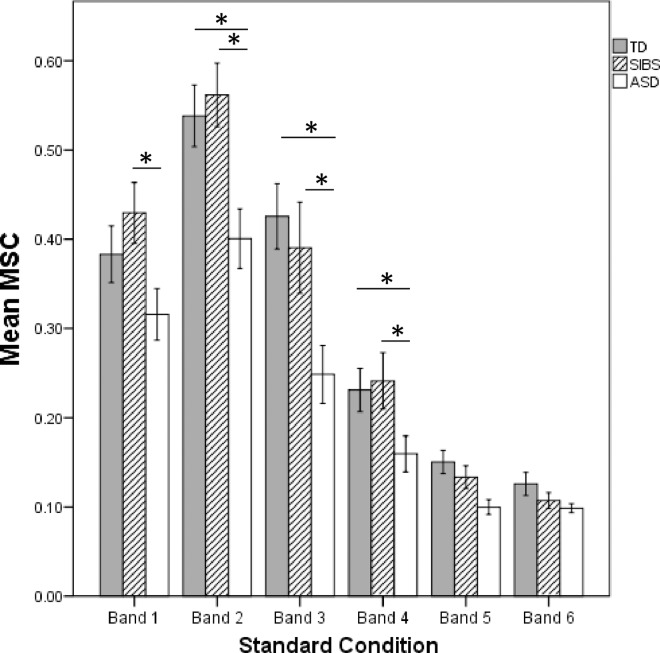
Mean magnitude squared coherence (MSC) by Group. Mean MSCs are displayed for the standard condition. Significance bars indicate p-values < .05. Error bars: +/- 1 *SE*.

There was a significant main effect by group for both the standard condition [F(12, 166) = 2.201, *p* = .014] and the short-duration condition [F(12, 172) = 2.008, *p* = .026]. For the standard condition, significant univariate main effects were obtained for Band 2 (*p* = .003), Band 3 (*p* = .003), Band 4 (*p* = .037) and Band 5 (*p* = .006). Similarly, for the short-duration condition, significant univariate main effects were obtained for Band 2 (*p* = .0468) and Band 3 (*p* = .001), and approached significance for Band 4 (*p* = .054) and Band 5 (*p* = .054). Pairwise comparisons indicated that results were driven by significantly weaker responses in these bands for the ASD group relative to TD and SIBS groups (Fig 3).

### Reliability between conditions

A two-level linear mixed-effects model was used to measure the absolute agreement across stimulus conditions within an individual. The covariance structure was variance components. Observer was treated as a random effect and stimulus condition was a fixed effect. Reliability of individual observers was strong for amplitude and latency measures with ICCs all > .85. ICCs for MSC bands were significant for Bands 1–5, with strong correlations for Bands 2, 3, and 4 (Table 3).

**Table 3:** Intraclass Correlation Coefficients (ICC) for Time- and Frequency-Domain Variables.

	ICC	*p*
P_60_-N_75_ Amplitude	.920	< .001
N_75_-P_100_ Amplitude	.889	< .001
N_75_ Latency	.873	< .001
P_100_ Latency	.892	< .001
Band 1	.445	.002
Band 2	.717	< .001
Band 3	.817	< .001
Band 4	.785	< .001
Band 5	.371	.010
Band 6	. 165	.183

Results show the consistency between the standard condition and short-duration condition. Frequency bands include the following: Band 1, 6–10 Hz; Band 2, 12–28 Hz; Band 3, 30–36 Hz; Band 4, 38–48 Hz; Band 5, 50–64 Hz, minus 60 Hz; Band 6, 66–84 Hz.

## Discussion

This study applied electrophysiological techniques to examine early-stage visual processing in children with idiopathic ASD and their unaffected siblings. Low-level visual stimuli were used to examine tVEP responses to a standard contrast-reversing checkerboard condition and a novel short-duration condition. The new stimulus condition was developed to improve objective data collection from severely affected or difficult to test populations, who are often excluded from EEG studies due to the cognitive, receptive language, or behavioral demands essential to many protocols. For this reason, children with ASD of varying levels of cognitive and adaptive functioning were included.

Results from time-domain analyses indicate that children with ASD display significantly smaller amplitudes than both TD children and SIBS for two of the earliest, critical VEP components, P_60-_N_75_ and N_75-_P_100_, which reflect primarily excitatory and inhibitory postsynaptic activity, respectively. These results add to a growing body of literature indicating reduced cortical activity in ASD [40–44]. The smaller P_60-_N_75_ amplitude found in the ASD group suggests weaker excitatory input to the cortex, which subsequently reduces the inhibitory component of the VEP. N_75-_P_100_ amplitude is a measure of the relative strength of inhibition to excitation. The loss of excitatory input seen in the ASD group was proportional to the loss of inhibitory input observed, which suggests that there is not a significant deficit in intracortical inhibition. If excitation was intact and decreased inhibition was present, we would expect significantly larger P_60-_N_75_ amplitudes. In this case, weaker input to the cortex reflects excitatory deficits, which subsequently result in less inhibition and an overall reduction in amplitude for both critical components.Response latencies did not differ among groups as all groups reached P_60_, N_75_, and P_100_ peaks and troughs within expected time intervals. These results indicate that there was no measureable delay in information getting to the visual cortex in the ASD group.

The greater loss in higher frequency activity in the ASD group may be due to the attenuated excitatory input to the cortex observed in this group. A loss in excitatory input is expected to lead to decreased intracortical synaptic activity and subsequent to that, reduced conductance across neuronal membranes, which would yield longer time constants and a loss in high frequency activity [45]. While our results suggest a loss of excitatory input at the cortical level, studies examining high frequency excitatory input to the cortex are currently underway to determine whether there is a high-frequency loss in cortical input or if the high-frequency loss is produced in the cortex.

Results from frequency-domain analyses provide additional support for decreased cortical responses in ASD. While time-domain analyses offer important information with regard to the magnitude and timing of responses, frequency-domain analyses are a more objective measure of the consistency of responses within different frequency components and bands. Our results indicate that children with ASD display greater response variability in bands encompassing both low (Band 3) and higher gamma-wave (Band 4) activity. Alterations in gamma oscillations within the visual cortex have been attributed to waveform abnormalities in clinical populations [46] and have been correlated with VEP responses, which may indicate that the neural populations generating these responses are the same [47]. In addition, although no studies have applied frequency-band analyses to tVEPs in ASD, these findings are consistent with EEG, magnetoencephalography (MEG), and functional magnetic resonance imaging (fMRI) studies describing abnormal gamma oscillations in several different brain regions [48–50], and may reflect some of the functional impairments observed in ASD. There is also literature describing the relationship between gamma-wave activity and GABAergic interneurons, including the potential role of gamma-band activity as an endophenotype that may be used to detect response to treatment [51]. While our VEP results may not reflect the same brain mechanisms examined when looking at ongoing, resting-state EEG or higher order visual stimuli, there could be overlap in visual responses and natural circuitry in the brain.

Future work applying stimulus conditions that measure nonlinear lateral and shunting inhibitory interactions can be used to explore the quality of specific inhibitory mechanisms in ASD [45, 52].Understanding the morphology of GABAergic neurons that are responsible for shunting inhibition versus those that play other inhibitory roles in the nervous system are important to better understand the E/I abnormalities found here, which suggest reduced inhibitory activity as a result of weaker excitatory input to the cortex.

With regard to the SIBS group, there were no differences in the amplitude or latency of responses relative to the TD group; however SIBS showed significantly stronger responses than their affected siblings and non-significant, but weaker responses than controls for response amplitudes. This pattern of intermediate brain activity is consistent with previous literature in unaffected siblings of individuals with ASD [53, 54]. Interestingly, there were no frequency domain differences between the SIBS and TD groups. This profile in which SIBS display intermediate amplitudes in relation to TD and SIBs groups with high reliability of responses requires further exploration into the potential role of an endophenotype.

The novel short-duration condition, which was created to enable data collection from severely affected or difficult to test populations, is a main contribution of this study. The short-duration condition allows for repeated trials with breaks as needed, requiring only three seconds of sustained attention per trial. Approximately 30 seconds of testing under this demonstrated comparable VEP data with respect to amplitude, latency, and early/mid-frequency band activity compared to a standard 60-s condition. In addition, data from the short-duration stimulus was collected from approximately 92% of children with ASD, compared to 68% for the standard stimulus. It is also notable that data collection was feasible from children with significant impairments in language and intellectual functioning. Given the critical need for objective outcome measures, the short-duration condition may be a helpful new tool for a variety of clinical disorders. In addition, consistent findings between the two stimulus conditions, specifically, the combination of smaller tVEP amplitudes and reduced frequency band activity may signify a diagnostic marker of ASD and warrants continued evaluation.

Overall, time- and frequency-domain analyses did not demonstrate a relationship between cognitive ability and age with VEP responses, suggesting that the differences observed here might be a marker of ASD broadly and, importantly, are not a consequence of intellectual functioning or development. However, given the heterogeneity of the disorder, further studies are needed in order to subgroup individuals based on neurophysiological profiles. In addition, while IQ is not expected to affect responses at such early stages of sensory processing, a comparison group of children with developmental delay and/or intellectual disability without ASD would enhance the generalizability of these findings. Future studies should examine VEPs longitudinally to determine whether developmental changes are present and whether there are differences in neural maturation between individuals with and without ASD. Finally, while there were significant differences among group means for the amplitude of VEP responses, analyses at an individual level are necessary for subtyping individuals with ASD based on excitatory/inhibitory (E/I) profiles. This type of subtyping may be an important future direction to determine appropriate treatment targets for a given individual. Future work should also include infant-sibs studies, which have the potential to offer important information on the utility of tVEPs as an early diagnostic marker of ASD while providing information on endophenotypes based on SIBS responses.

In the long-term, VEPs may be useful for clinical trials and testing the efficacy of medications that affect glutamatergic or GABAergic systems. Baseline VEP responses could be used as inclusionary criteria (e.g., choosing participants with weak excitatory responses) and then applied as an outcome measure or a method to gather information on change and possibly optimal drug dosage. The results of the current study may provide some explanation for the failure of many clinical trials in ASD, as the heterogeneity of the disorder requires personalized approaches and objective outcome measures.

Overall, VEPs are advantageous as they are a rapid, reliable, and well-understood method to gather information about postsynaptic brain activity. VEPs can be obtained using only a single electrophysiological channel (three electrodes), as was done in this study, and have been used on infants in the first few weeks of life [55–57]. In addition, a Current Procedural Terminology (CPT) code for VEPs already exists, adding to the potential clinical utility of these methods. Longitudinal natural history studies are needed to determine when abnormalities emerge and whether they are present throughout the lifespan. Correlating neural findings from diffusion tensor imaging (DTI) studies of the optic nerve as well as magnetic resonance spectroscopy (MRS) studies examining GABA levels in the visual cortex are also essential next steps.

The current study establishes the utility of a short-duration tVEP test for use in children with ASD at varying levels of functioning. In addition, specific deficits were observed including reduced amplitudes at the two earliest critical peaks in the tVEP waveform and weaker responses in gamma-band activity, which play an important role in balanced excitation and inhibition. The methods used in this study may be integrated into future clinical trials to assess the efficacy of VEPs as a measure of change in response to treatment or a predictor of treatment response. This is the first known study to examine VEP markers in a sample inclusive of children with comorbid intellectual disability and behavioral challenges, while establishing the utility of a novel short-duration VEP test for use in this population.
